# All dielectric metasurface based diffractive neural networks for 1-bit adder

**DOI:** 10.1515/nanoph-2023-0760

**Published:** 2024-01-24

**Authors:** Yufei Liu, Weizhu Chen, Xinke Wang, Yan Zhang

**Affiliations:** Beijing Key Laboratory of Metamaterials and Devices, Key Laboratory of Terahertz Optoelectronics, Ministry of Education, Beijing Advanced Innovation Center for Imaging Theory and Technology, Department of Physics, Capital Normal University, Beijing, 100048, China

**Keywords:** diffractive neural network, metasurface, optical computing

## Abstract

Diffractive deep neural networks (*D* ^*2*^ *NNs*) have brought significant changes in many fields, motivating the development of diverse optical computing components. However, a crucial downside in the optical computing components is employing diffractive optical elements (DOEs) which were fabricated using commercial 3D printers. DOEs simultaneously suffer from the challenges posed by high-order diffraction and low spatial utilization since the size of individual neuron is comparable to the wavelength scale. Here, we present a design of *D* ^*2*^ *NNs* based on all-dielectric metasurfaces which substantially reduces the individual neuron size of net to scale significantly smaller than the wavelength. Metasurface-based optical computational elements can offer higher spatial neuron density while completely eliminate high-order diffraction. We numerically simulated an optical half-adder and experimentally verified it in the terahertz frequency. The optical half-adder employed a compact network with only two diffraction layers. Each layer has a size of 2 × 2 cm^2^ but integrated staggering 40,000 neurons. The metasurface-based *D* ^*2*^ *NNs* can further facilitate miniaturization and integration of all optical computing devices and will find applications in numerous fields such as terahertz 6G communication, photonics integrated circuits, and intelligent sensors.

## 1 Introduction

Diffractive deep neural networks (*D* ^*2*^ *NNs*) [1] constitute a burgeoning all-optical machine learning framework, processing task-specific information modulation via the manipulation of input coherent light fields via a successive passive diffractive layers. These layers comprised of numerous individual phase and amplitude modulation units and multiple free-space propagation. With appropriate phase and amplitude distribution of each layers, *D* ^2^ *NNs* can fit arbitrary complex-valued linear transformations between input field-of-view and output field-of-view [2], [3], [4]. The phase or amplitude distribution of each layer can be inversely optimized by conventional deep learning methods such as stochastic gradient descent and error back-propagation. *D* ^2^ *NNs* have been expected to be an alternative to electronic systems as a task-specific computing device owing to its advantages in power efficiency, low latency, and parallelization capabilities. They have been demonstrated remarkable performance in various applications, including object recognition [1], [5], [6], [7], [8], spectrum control [9], [10], and logical computing [11], [12]. Recent researches has explored ways to enhance the complexity and information processing capabilities of *D* ^2^ *NNs* by introducing polarization [13], wavelength multiplexing [14], and optoelectronic networks [6], [15], [16]. However, the basic unit of each layer employs the diffractive optical elements (DOEs) which are fabricated with commercial 3D-printers, the scale of these conventional thickness-based modulation units for phase and amplitude modulations are comparable to the wavelength which limites the potential of miniaturization and integration of *D* ^2^ *NNs*, thus this kind of nets suffers the drawbacks of low resolution and low efficiency due to the introduction of high-order diffraction.

Metasurface is a kind of two-dimensional artificial electromagnetic material, which consists of subwavelength-scale optical microstructure units also named optical antennas [17], [18], [19]. It can be used to arbitrarily modulate the properties of the scattered wave, including frequency, amplitude, phase, and polarization, based on the electromagnetic resonances excited by electromagnetic waves and periodic or non-periodic arranged optical antennas. The specific values of each modulation parameter can be adjusted within a considerably wide range by changing the geometrical parameter of the optical antennas, e.g., shape, size, or arrangement. With its unique characteristics of high integration, free from high-order diffraction, and high modulation degrees of freedom, metasurface has distinct advantages in optical information processing field. In recent years, metasurfaces have been used to archive various functions, such as holography [20]–[26], special beam generation [27]–[31], and polarization modulation [32]–[36]. For resent years, artificial intelligence (AI) and deep learning methods has been introduced into metasurfaces, improving the flexibility of metasurface devices [37], [38], [39], [40].

**Figure 1:**
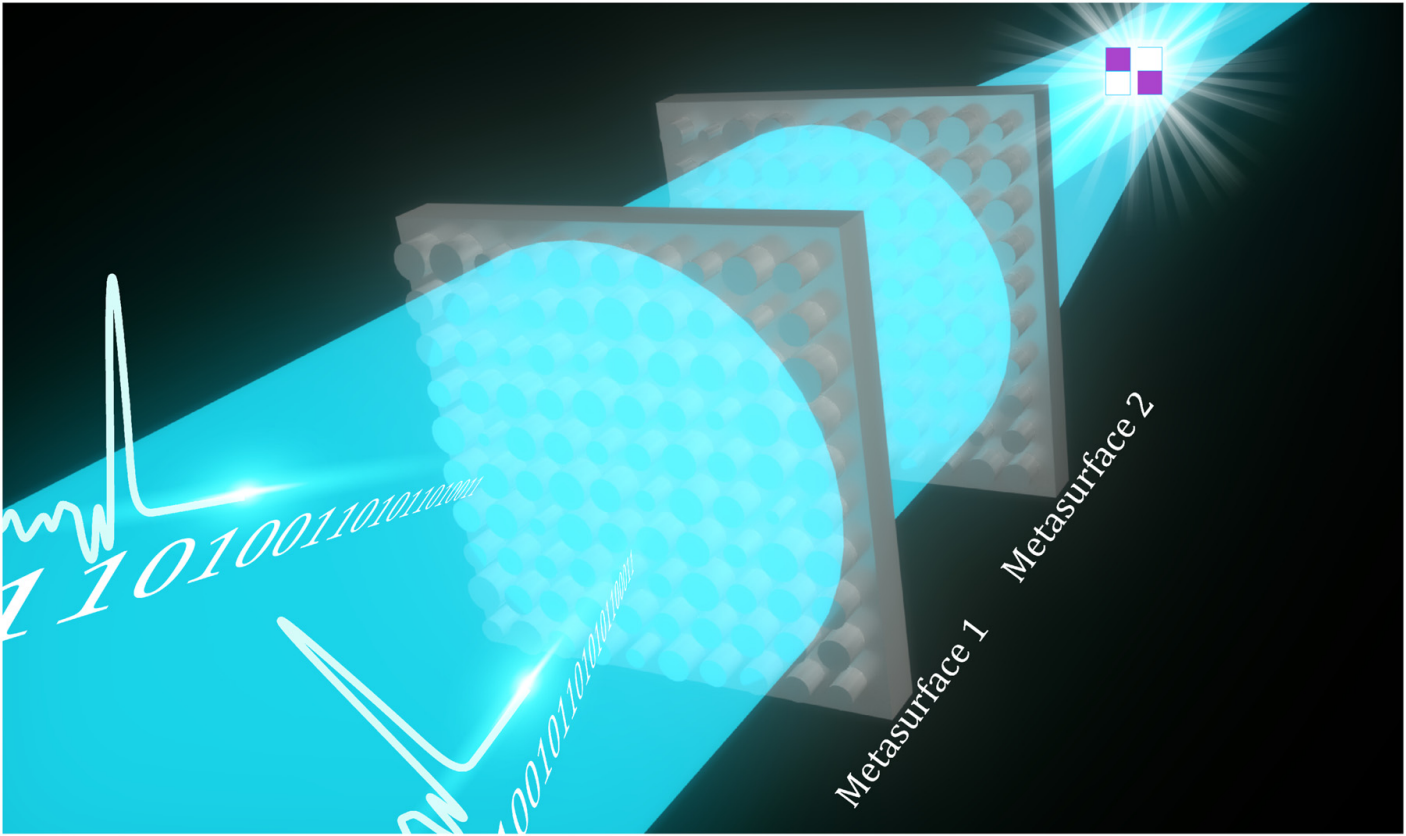
Schematic of metasurface-based *D* ^2^ *NN* for 1-bit half adder, which accepts binary optical signal as input and produces the spatially encoded results.

In this paper, we will introduce metasurfaces into *D* ^2^ *NNs* and design an optical half-adder in the terahertz frequency range to experimentally verify our approach. As shown in Figure 1, all dielectric silicon cylinder metasurfaces are used as diffractive layers. The diameter value of each cylinder in the metasurfaces, simulated using a finite-difference time-domain (FDTD) algorithm, corresponds to the required pure phase modulation value in the range of 0 to 2*π* after 16-level quantization. The phase distributions of layers are numerically optimized via conventional *D* ^2^ *NNs* methods (i.e., stochastic gradient descent and error back-propagation) in a computer. The metasurface-based *D* ^2^ *NNs* can be more compact and more efficient than conventional terahertz DOEs networks, escaping from the restrictions of high-order diffraction and fabrication precision. 40,000 neurons (micro-cylinders) are integrated into each metasurface layer with a spatial size of only 2 × 2 cm^2^ where each micro-cylinder has a size of 100 × 100 μm^2^ is significantly smaller than the working wavelength (353 μm). The final results demonstrate that our design can achieves powerful computing capability comparable to the state-of-the-art DOE-based *D* ^2^ *NNs*. The metasurface-based *D* ^2^ *NN* can further facilitate miniaturization and integration of all optical digital computing components. It is suitable to be implemented on space-constrained computing applications, e.g., 6G communication, photonics integrated circuits, and intelligent sensor. The metasurface can endow new degrees of freedom to *D* ^2^ *NN*. It is believed that the metasurface-based *D* ^2^ *NNs* can provide a robust and innovative framework for all optical computing, and explore a new direction for optical neural networks. With future complete utilization of unique property of metasurface, such as high integration, polarization controlling, and optical non-linear, this framework will serve as a potent engine for development of all optical computing devices.

## 2 Results

**Figure 2:**
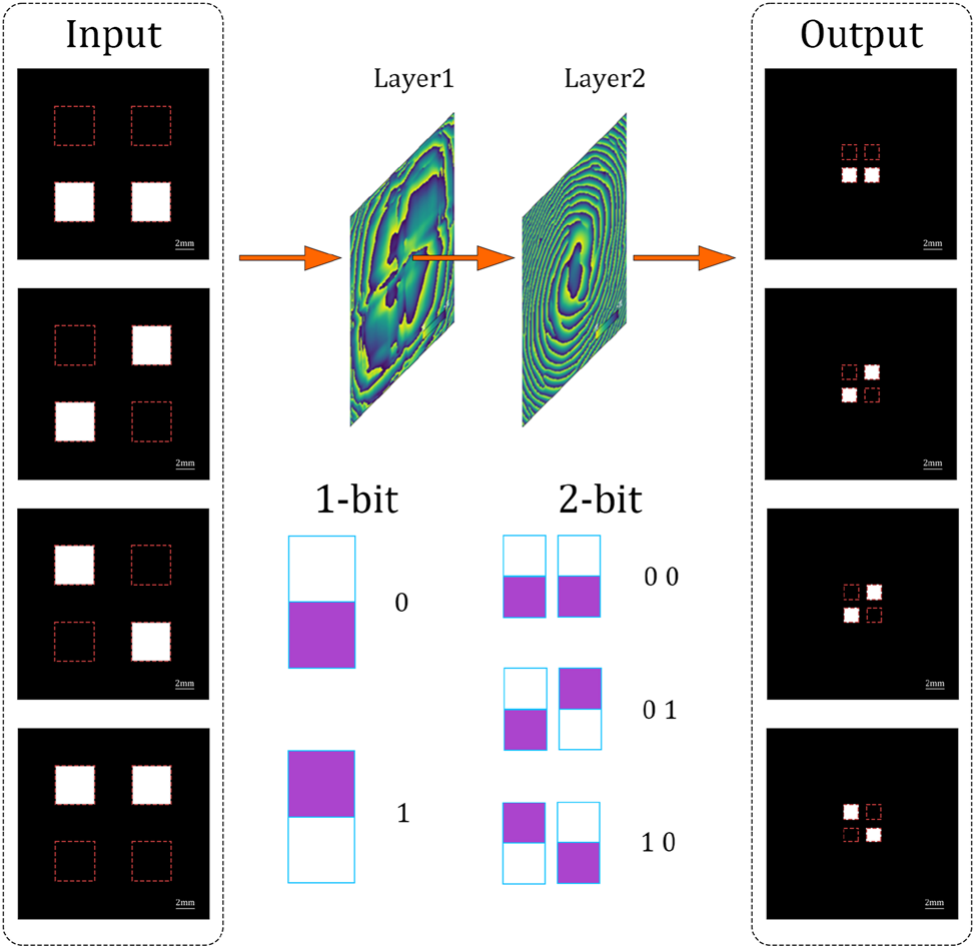
Architectonics and encoding method for the metasurface-based half-adder. Two layers of the metasurface-based half-adder receive two 1-bit spatial intensity signals and generate a 2-bit spatial intensity output signal while both input and output are encoded with the distribution of light intensity within special ranges. Input apertures are set to 4 × 4 mm^2^ which are larger than the output apertures (1.6 × 1.6 mm^2^) to utilize more energy.

To perform the addition computing task with optical network, the input and output signals are encoded in spatial intensity distributions, as shown in Figure 2. The 1-bit information is encoded by a pair of square apertures, similarly to the previous work [12]. The decoded binary number is 0 or 1 is determined by the relative power of upper or lower apertures. If the relative power in the upper aperture is higher than 50 %, it means the binary number is 1, else if the relative power in the lower aperture is higher than 50 % the binary number is 0. The 2-bit output pattern is the combination of two 1-bit patterns. Both input and output planes have four designed square areas to carry information. Input apertures are set as 4 × 4 mm^2^ which are larger than the output apertures (1.6 × 1.6 mm^2^) to utilize more energy for computing. The expanded input aperture can increase the input energy and improve the signal-to-noise ratio of the results.

**Figure 3:**
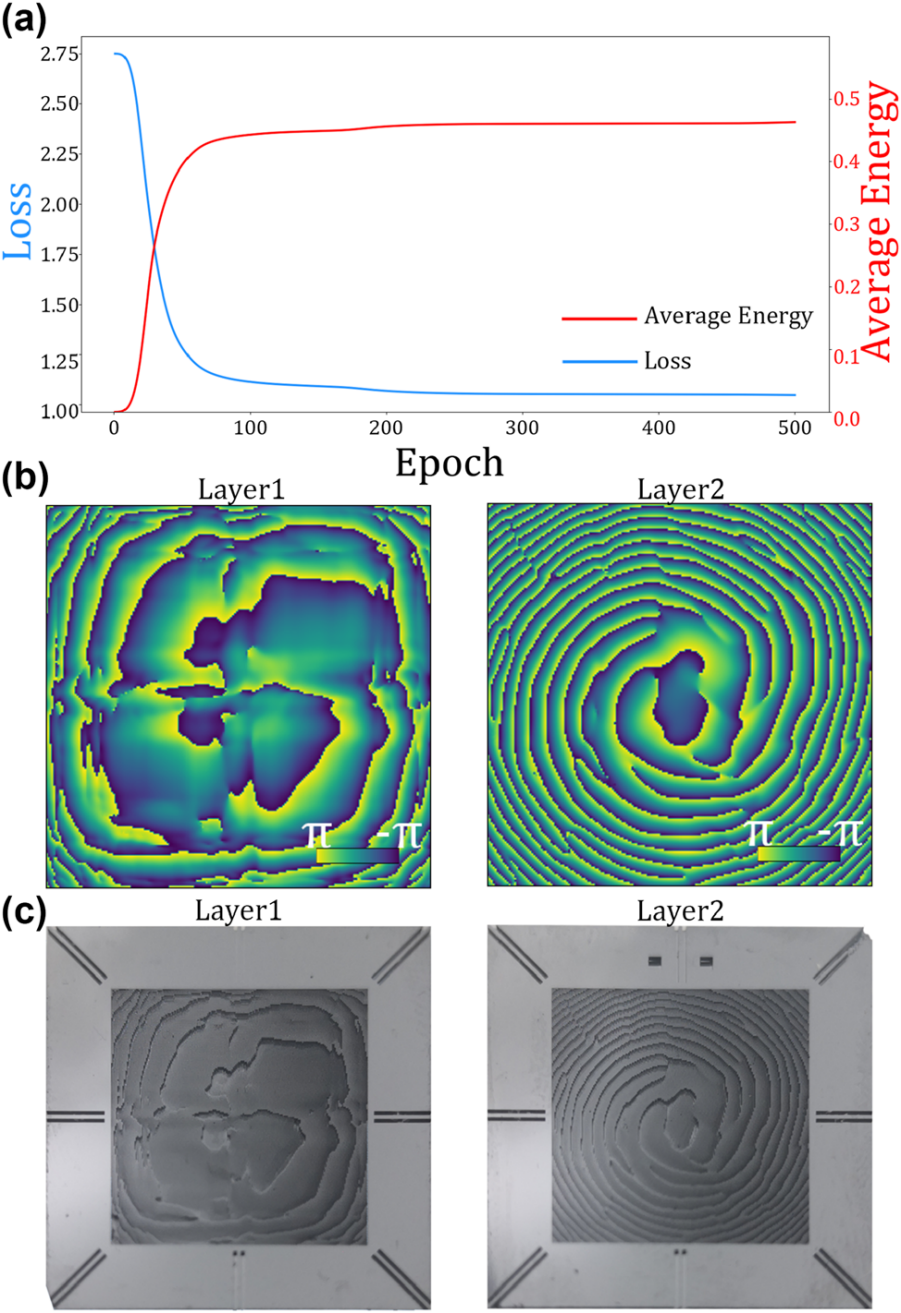
Optimized results of the metasurface-based half-adder. (a) Dependence of loss and energy on the training epochs. (b) Optimized phase distributions of two layers. (c) Physical images of two layers fabricated on silicon substrates.

To suit the space-constrained application requirement, a two-layer network has been trained for the 1-bit half-adder. Every neurons are optimized during iteration of epoch using standard deep learning tools after random initialization. The loss value is calculated from the output of each forward propagation using the MES loss function (refer to Appendix A for further details) and a backward propagation is performed using the Adam optimizer subsequently. The curves of the loss and corresponding average energy efficiency are shown in Figure 3(a). It can be observed that gradients tend to approach zero as the iterations increase, indicating the training has converged and most of the energy has been concentrated in the desired square apertures. To indicate the degree of the energy is concentrated into the correct regions, the average energy (AE) is defined as:

(1) $$AE=\frac{1}{4I_{i}}\overset{4}{\underset{1}{\sum }}\left(I_{b1}+I_{b2}\right),$$

where *I*_*i*_ is the total energy on the input plane, which is a constant for the simulation program. $\frac{1}{4}\sum_{1}^{4}$ represents the calculating the average energy of all four output, *I*_*b*1_ and *I*_*b*2_ are the energy of the first and second bit in its correct area, represented by:

(2) $$I_{b1,b2}=\underset{n\in \text{right}\;\text{area}}{\sum }{\left|E_{n}\right|}^{2},$$

where *E*_*n*_ is the electric field of the *n*th pixel on the output plane.

As a result, two phase distributions have been optimized, as depicted in Figure 3(b). The phase values of 200 × 200 neurons are warped into the ranges of −*π* to *π*. The metasurface are fabricated on silicon substrates where the neurons is expressed with the corresponding micro-cylinders designed by the FDTD simulation after 16-level quantization (refer to Appendix A for further details). The photos of fabricated metasurfaces are shown in Figure 3(c). The metasurfaces with the size of 2 × 2 cm^2^ are placed at the central position of silicon substrates. There are some etch lines at the edges for alignment, and the similar structure have also been used in previous research [41]. The distance between the diffractive layers is set as 10 cm and the distances between the input plane to the first layer and the second layer to the output plane are selected as 2 cm. Several measurements are carried out to mitigate against the misalignment error among the input plane, diffractive layers, and output plane.

**Figure 4:**
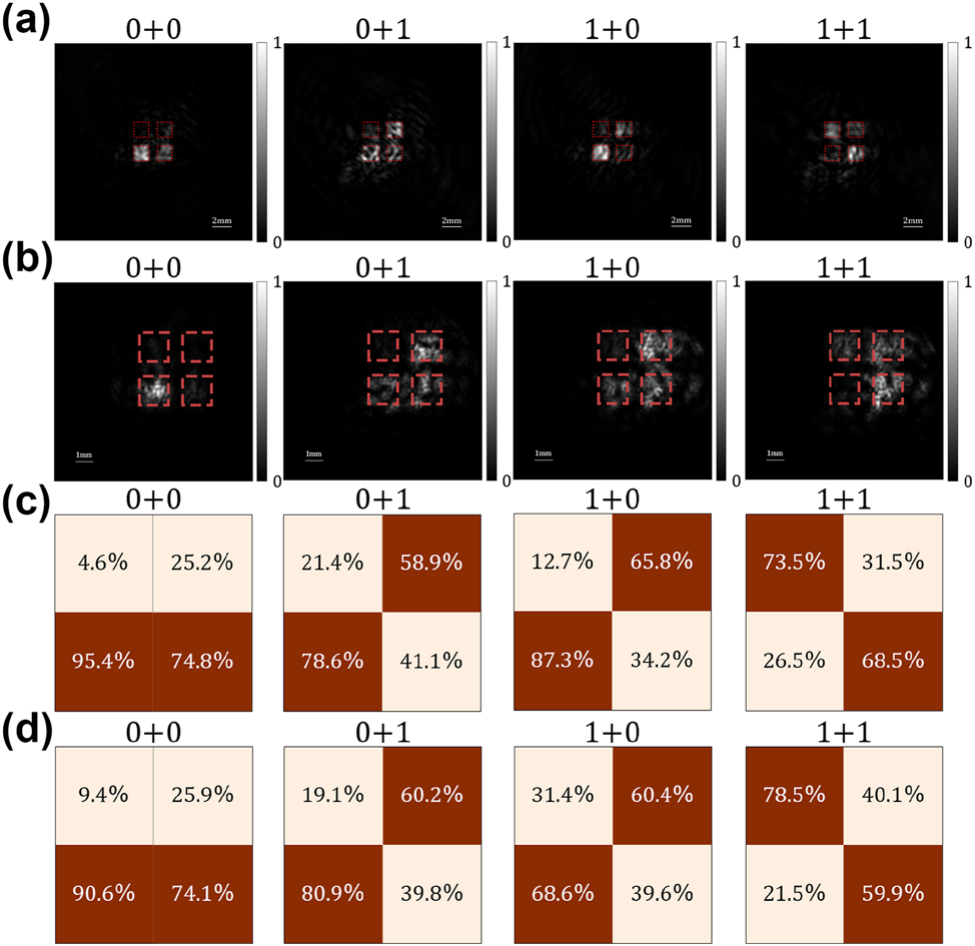
Simulation and experiment results of the metasurface-based half-adder. (a) The simulation results of intensity distribution on the output plane, (b) the experiment results of intensity distribution on the output plane, (c) the energy distribution of simulation result, and (d) the energy distribution of experiment result.

The numerical and experimental results are shown in Figure 4, including overall 4 kinds of possible input of the half-adder. The simulated and experimental intensity distributions on the output plane are presented in Figure 4(a) and (b), respectively. The four apertures, which carry information, are marked by the red-bordered square. The value of binary number is determined by the relative power in the upper and lower red-bordered areas. Through pixel-by-pixel summing and comparison, the decoded binary results and energy distributions of four designed regions are depicted in Figure 4(c) and (d), where the energy distributions of up aperture (*E*_up_) and down aperture (*E*_down_) are calculated as:

$$E_{\text{up}}=\frac{I_{\text{up}}}{I_{\text{up}}+I_{\text{down}}},$$

(3) $$E_{\text{down}}=\frac{I_{\text{down}}}{I_{\text{up}}+I_{\text{down}}},$$

where *I*_up_ and *I*_low_ represent the total intensity in the upper and lower square aperture, respectively.

The decoding processing from spatial intensity information to binary numbers is equivalent to performing a binarization of energy distribution with a threshold of 50 %. The darker orange squares in Figure 4(c) and (d) represent higher energy comparing to the other aperture in the same bit pattern, i.e., correct output aperture for this bit. Benefit from the enough contrast between the energy concentrated in the different apertures, the computing results can be recognized and decoded easily, which indicates the proposed half-adder can implements the optical addition computing well, like the state-of-the-art DOE-based *D* ^2^ *NN*.

## 3 Discussion and conclusions

In conclusion, we have successfully introduced the metasurface into *D* ^2^ *NNs*, taking a significant stride towards the advancement of all optical computing technology. The diffractive layers in the proposed network are adopted with all-dielectric cylinder metasurfaces, which are not only compact and thin but also can overcome the limitations of conventional DOE, such as high-order diffraction and low spatial utilization. An optical half-adder is demonstrated in the terahertz frequency range. However, the volume of optical computing system is not only determined by diffractive layers, but also relies on the distance between each device. To mitigate against the misalignment error, the distance between diffractive layers of half-adder is still relatively large which increases the volume of optical computing system, which can be decreased by using more precise holder or training a misalignment resilient network [42].

This work successfully establishes a compact and powerful all optical computing framework. It validates the feasibility of transplanting *D* ^2^ *NNs* to metasurface. The design just utilize one of characteristics of metasurface, i.e., high integration, more characteristic of the metasurface, e.g., polarization controlling, direction choosing, and optical non-linear effect, can be concentrated to increase the degree of freedom of *D* ^2^ *NNs*. The metasurface-based *D* ^2^ *NNs* have immense potential to apply in space-constricted computing applications such as 6G communication, photonic integrated circuits, and intelligent sensors.

## Figures and Tables

**Figure 5:**
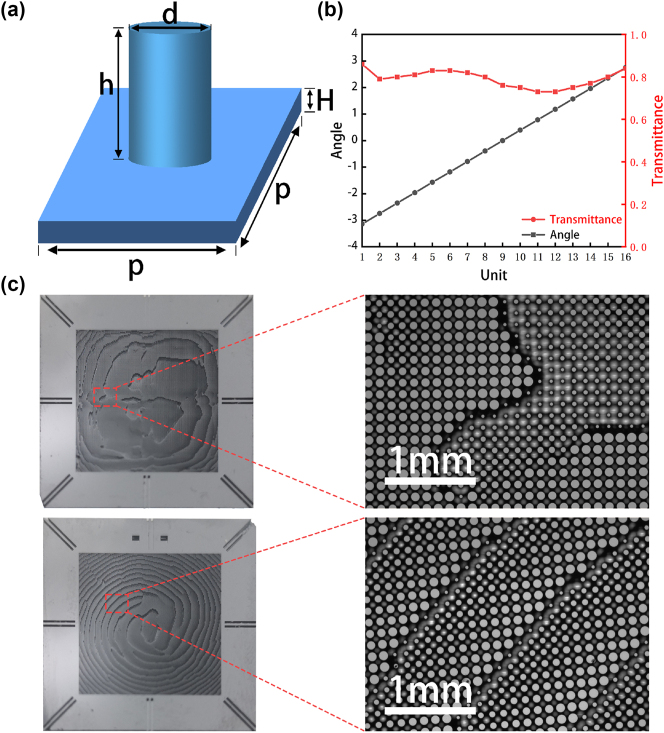
Schematic of neurons and metasurfaces. (a) Geometrical schematic of neurons (modulation unit), where the cylinders with a height *h* of 240 μm and 16 kinds of diameters ranging from 30 to 86.97 μm periodic are located on the substrate of silicon with a period *p* of 100 μm. The thickness of *H* is 760 μm. (b) Amplitude and phase modulation of neurons with 16-level diameters for working frequency of 0.85 THz. (c) Microscopic images of two fabricated metasurfaces.

**Figure 6:**
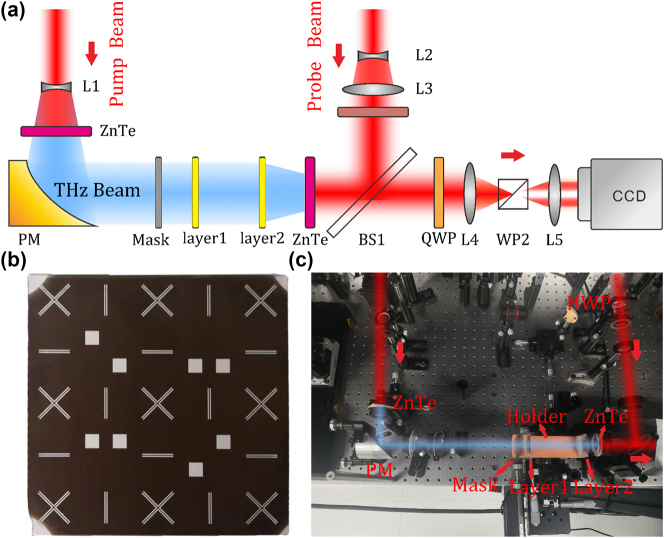
Experiment setup for metasurface-based 1-bit adder. (a) 2D schematic diagram of experimental system. L, lens; PM, parabolic mirror; HWP, half-wave plate; BS, beam splitter; QWP, quarter-wave plate; WP, Wollaston prism; and CCD, charge-coupled device. (b) Optical mask for generating THz intensity distributions of input signal. (c) Optical image of experimental system.

**Figure 7:**
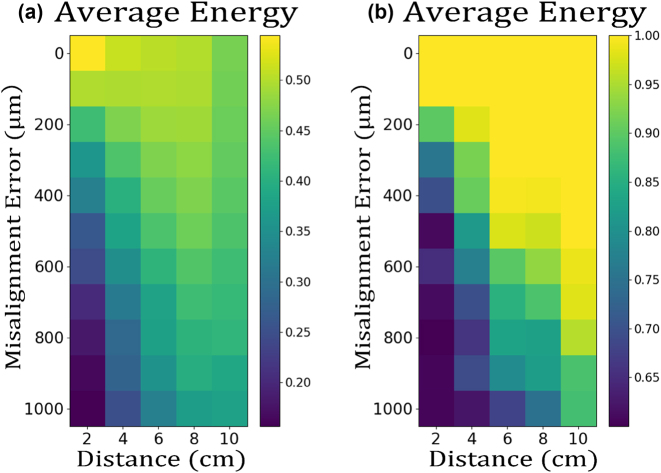
Dependences of average energy (a) and computing accuracy (b) of the optical adder with different diffractive distances on the random misalignment.

**Figure 8:**
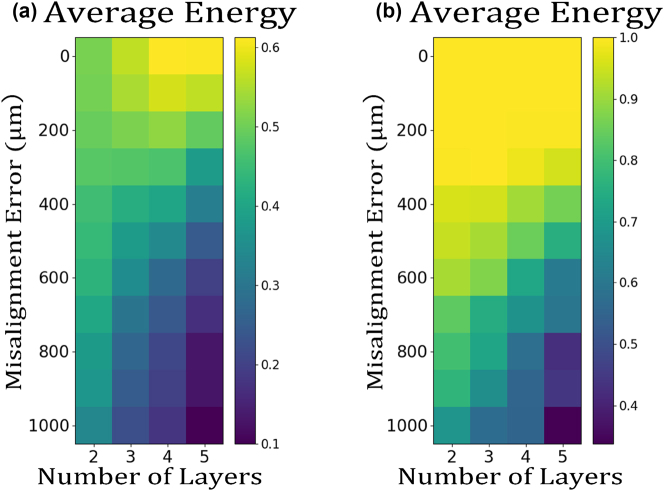
Dependences of average energy (a) and computing accuracy (b) of the optical adder with different number of layers on the random misalignment errors.

**Figure 9:**
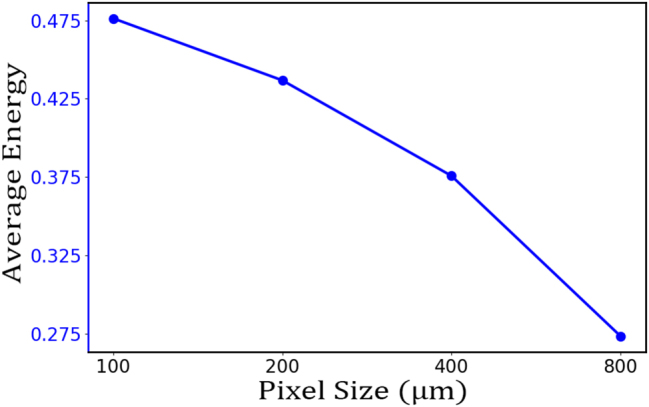
Influence of the pixel sizes. The average energy is continuously decreasing with the pixel size increases.

## Appendix A: Materials and methods

### A.1 Forward-propagation model

In the design, the *m*th modulation unit (neuron) in the *l*th diffractive layer can be represents a complex-valued transmission modulation, *t* ^*l*^, given by:

(4)
$$t^{l}\left(x_{m},y_{m},z_{m}\right)=Ae^{j\phi^{l}\left(x_{m},y_{m},z_{m}\right)},$$

where *A* represents a constant amplitude transmission coefficient, and *ϕ* ^*l*^(*x*_*m*_, *y*_*m*_, *z*_*m*_) is the phase modulation with a value between 0 and 2*π* of the *m*th neuron, it is a trainable parameter.

The output optical field of *m*th unit on the *l*th diffractive layer can be represented by the complex-valued modulation of the optical field of (*l*–1)th layer [1], [13] as

$$E^{l}\left(x_{m},y_{m},z_{m}\right)=t^{l}\left(x_{m},y_{m},z_{m}\right)\cdot \overset{N_{0}}{\underset{n=1}{\sum }}[E^{l-1}\left(x_{n},y_{n},z_{n}\right)\cdot$$

(5)
$$w_{m}^{l}\left(x_{m},y_{m},z_{m}\right)],$$

where *N*_0_ is the total number of neurons on the (*l*–1)th layer, $w_{m}^{l}\left(x_{m},y_{m},z_{m}\right)$ denotes the free-space wave propagation modeled through the Rayleigh–Sommerfeld diffraction equation:

(6)
$$w_{m}^{l}\left(x_{m},y_{m},z_{m}\right)=\frac{z_{l}-z_{l-1}}{r^{2}}\left(\frac{1}{2\pi r}+\frac{1}{j\lambda }\right)e^{\frac{j2\pi r}{\lambda }},$$

where *λ* denotes the working wavelength, *z*_*l*_ and *z*_*l*−1_ are the positions of *l*th and (*l*–1)th layer, *r* is the distance between the *m*th unit located at (*x*_*m*_, *y*_*m*_, *z*_*m*_) of *l*th layer and the *n*th unit located at (*x*_*n*_, *y*_*n*_, *z*_*n*_) of (*l*–1)th layer.

### A.2 Loss function

For the all optical half-adder, the mean squared error (MSE) is employed as loss function while error back propagating

(7)
$$L_{\text{MSE}}=\frac{1}{N}\overset{N}{\underset{n=1}{\sum }}{\left|O_{n}-{\hat{O}}_{n}\right|}^{2},$$

where *O*_*n*_ denotes the optimized output intensity distribution on the *n*th pixel and ${\hat{O}}_{n}$ is the target (ground truth) output intensity distribution. *N* is the total number of pixels on the output plane. The loss for one of four inputs can be calculated with Eq. (7). The optimization target is to minimize the average loss $\left(L_{\text{Avg}}\right)$ for all 4 kinds of input:

(8)
$$L_{\text{Avg}}=\frac{1}{4}\overset{4}{\underset{1}{\sum }}L_{\text{MSE}},$$

### A.3 Training details

The training is implemented using Python version 3.10 and Pytorch framework version 1.12.1. The angle spectrum method is employed to calculate forward propagation. The detailed parameters of training are shown in Table 1.

**Table 1:** Parameters used in training.

Training parameter	Value
Epochs	500
Batch size	4
Learning rate	1.2e-2
Optimizer	Adam
Loss function	MSE

The training curve, as shown in Figure 3(a), is affected by various factors. When the training is totally convergence (the curve is nearly a horizontal line), the value of the loss and average energy is stable. The final loss and energy are determined by the physical parameters of the system, for example, the number of layers, diffractive distance between the layers, and design of output plane. When the training is not convergence, the training parameters will affect the speed of convergence, whether the training will convergence and whether the result is globally optimal solution. The training parameters, such as batch size, learning rate, and training epoch, have been repeatedly adjusted in the design.

### A.4 Design of neurons

The schematic diagram and property of neurons for the metasurface are depicted in Figure 5. The metasurfaces are fabricated in the substrate of high-resistivity silicon (thickness = 1 mm). The geometrical schematic of a neuron is shown in Figure 5(a). The period of neuron, *p*, is set as 100 μm. The 240 μm height cylinder, fabricated through ion beam etching process, is located at the center of the neuron. Figure 5(b) shows the amplitude and phase transmittance of neuron changed with the diameter *d* of cylinder after 16-level quantization. The maximum diameter is 86.97 μm while the minimum diameter is 30 μm. It is can be observed that as the value of phase modulation increases from −*π* to *π*, the amplitude modulation is maintained around 0.8. The microscopic images for two physical metasurfaces are shown in Figure 5(c).

### A.5 Experiment setup

The experiment is implemented on a standard terahertz focal plane imaging system, as depicted in Figure 6. Figure 6(a) illustrates a schematic diagram of the system. The light generated from a femtosecond pulse laser, is divided into two beams: The pump beam is used to generate terahertz wave, and the probe beam to record the terahertz wavefront. The charge-coupled device (CCD) can transfer the information from optical signal to electrical signal. To generate the desired input intensity signal, a chromium-plated mask as shown in Figure 6(b) has been used to block the terahertz beam and to generate the spatial intensity distribution. Since the pixel size is reduced, aligning the diffractive layers has become a serious issue. The transverse moving of input mask relative to the metasurface will only result a shift of output pattern on the detection plane, which will not affect the result. However, the misalignment of diffractive layers will cause the confusion of output pattern. In order to reduce this misalignment error in the experiment, a holder shown in Figure 6(c) is manufactured using a 3D printer, the meatsurfaces are fixed in the holder. Some marks are etched along with structures, as shown in Figure 3(c), for better alignment.

## Appendix B: Misalignment error analysis

The number of layers and the distance between diffractive layers will affect the robustness of the system. The numerical simulations are carried out to aid us to find the better parameters. The same input is fed into the diffractive network, a random shift between adjacent diffractive layers is used to simulate the misalignment error. Figure 7 presents the variation of energy efficiency and accuracy with respect to the distance and misalignment. It can be seen that the energy focused in the correct area and the accuracy of the output will inevitably decrease as the misalignment error increases from 0 to 1000 μm. However, the impact of misalignment declines as the distance between two metasurface layers increases. When the diffractive of a pixel on the previous layer cannot cover all pixels on the next layer, the system becomes more sensitive to misalignment errors. Considering the information loss with increasing diffraction distance, a compromise distance of 10 cm is selected in the experiment.

The effect of layer numbers on the performances and robustness of system is also investigated numerically. The reasoning capability and the information processing capacity will increase as the number of layers increases, had been proved in previous research [3]. However, the average energy and accuracy will significantly decrease with misalignment errors for multi-layer system, as shown in Figure 8, which will bring additional challenges to the experiment. Considering the space-constricted target application, the performance of a two-layer adder is sufficient to complete the computing task.

## Appendix C: Influence of the pixel size

As mentioned in the main text, the pixel size of our metasurface adder can reach one-third of the wavelength (100 μm compare to 352 μm). The smaller pixel size of our optical adder brings the following advantages compare to the previous researches: (1) A thin, slight optical computing device can be deeply integrated and embedded into a miniaturized computing system. (2) Due to the high-order diffraction, the stray light will appear around the target aperture when the pixel size is significantly larger than the working wavelength. Figure 9 provides a calculation on the diffraction efficiency varied with the pixel size while the size of layers and the diffractive distance are kept as constants. It can be seen that average energy decreased with pixel size increases. (3) The pixel size in this work is one-third of working wavelength (100/352). Comparing to the previous research (400/750) [1], the axial length between the layers can be compressed even the full-connection between diffractive layers should be satisfied. (4) Although only the phase-modulation of the metasurface has been used in this work, new degree of freedom, such as amplitude and polarization modulations, can be adopted to design *D* ^2^ *NNs* with more functions.
